# Supplementary material for: Hepatitis C (HCV) among Black and Latino sexual minority men (SMM) in the Southern United States: Protocol of a prospective cohort epidemiological study
Source: PLoS One. 2023 Jul 6;18(7):e0288129. doi: 10.1371/journal.pone.0288129 (PMC10325100; doi:10.1371/journal.pone.0288129)
Supplement: Attachment — Submitted filename: PLOS-ONE-09423R1.pdf [file pone.0288129.s001.pdf]

Manuscript Number: PONE-D-22-09423R1
Article Type: Study Protocol
Title: Hepatitis C Among Black and Latino Sexual Minority Men in the Southern United States: Protocol of a Prospective Epidemiological Study

The manuscript discusses a study protocol that seeks to inform HCV clinical guidelines (i.e., effective strategies for HCV screening among Black/Latino SMM), intervention development, prevention and treatment activities and development of patient assistance programs for the treatment of HCV among uninsured persons in the U.S. Deep South.

Throughout, the authors discuss the employment of a prospective cohort epidemiological study of HCV among Southern U.S. Black and Latino sexual minority men. The proposed study sample would include 400 Black and Latino SMM, aged 18 years and above. At the time of manuscript submission, 92 participants had been enrolled in study at the Washington D.C. site.

Introduction. The manuscript includes a strong introduction and literature review. The epidemiological data presented is clear, timely, and nuanced. In particular, the authors note the dearth of current scholarship focused on HCV burden among intersectionally marginalized communities, such as Black and Latino sexual minority men. Overall, both the introduction and literature review provide solid justification for the proposed study protocol and empirical study of HCV among HIV-negative Black and Latino SMM.

Methods: *Study sites and target population.* While the methods section, including the subsection 'Study Sites and Target Population,' is well-crafted, in describing the ongoing collaborative partnership between the two key community-based, non-profit organizations, the authors should describe how their partnerships with the community organizations were facilitated. Presently, this is unclear.

Methods: *Sample Size*. In detailing the proposed protocol study's research design, the authors should provide an empirical rationale for the proposed study sample size (N = 400). At present, no clear justification is provided.

Methods: *Inclusion/Exclusion Criteria: Age*.
The reviewer strongly suggests that the authors provide an empirical rationale for the stated inclusion/exclusion criteria related to age (i.e., 18 years and up). Indeed, 18-years and over encompasses a range of developmental stages that may be related to HCV incidence and/or prevalence among the population of interest (BLSMM). As mentioned, please provide an empirical rationale for this methodological relative to other studies that focus on particular age groups, e.g., ages 13 to 24; 18 to 25; 30 and up. Given that the manuscript is a study protocol, it is important for the authors to unpack their methodological decision-making in its entirety.

Methods: *Overview of Study Visits*. Notably, the reviewer appreciates the careful attention paid to unpacking this component of the proposed research protocol (i.e., Initial study visit, Integrated STI screening, Study questionnaire, Instruments, Translation of study materials, analysis plan, data management, quality assurance, & control, and statistical analysis). Important given the outcomes and study population.
