# Supplementary material for: Hepatitis C (HCV) among Black and Latino sexual minority men (SMM) in the Southern United States: Protocol of a prospective cohort epidemiological study
Source: PLoS One. 2023 Jul 6;18(7):e0288129. doi: 10.1371/journal.pone.0288129 (PMC10325100; doi:10.1371/journal.pone.0288129)
Supplement: Attachment — Submitted filename: Response to Reviewer Comments.docx [file pone.0288129.s002.docx]

March 28, 2023

Dr. Emily Chenette

Editor-in-Chief

PLOS ONE

Dear Dr. Chenette:

We are pleased with the opportunity to resubmit our manuscript, *Hepatitis C (HCV) among Black and Latino sexual minority men (SMM) in the Southern United States: Protocol of an epidemiological study, in consideration for publication as a protocol article in PLOS ONE.* We believe the manuscript, in its’ current state is ready for publication.

We appreciated the thoughtful comments of the reviewers from our initial submission

On the page that follow, we include a table that outlines the reviewer comments and our corresponding responses. In the revised manuscript, changes are highlighted.

Please do not hesitate to contact me if you have any questions or require further information. Thank you for your consideration and we look forward to your response.

Adedotun Ogunbajo, PhD, MPH

**Reviewer #3 Comments**

| **Comments** | **Response** |
| --- | --- |
| In general, I recommend not using the term "Blacks", instead using "Black populations", "Black participants", "Black individuals", etc. | We appreciate this comment and have made a global change to reflect this. |
| Have any participants been recruited at the Texas site? Only the number for the DC site is currently specified. | These numbers have now been updated to reflect the most up to date number of recruited participants. |
| I recommend a brief sentence in the discussion on how the recruitment limitations (i.e., excluding Black and Latino SMM who don’t receive health services) may potentially bias results (such as leading to an overestimate of healthcare-access related factors). | We have now included this in the revised manuscript. |
| I recommend adding the focus on an important minority population for HCV prevention/treatment efforts as a strength. | We appreciate this comment and have now added this important point |

**Reviewer #4 Comments**

| **Comments** | **Response** |
| --- | --- |
| The study fails to address how 400 participants is large enough to answer the research question and to draw valid conclusions. The authors should rewrite this section and include the sample size calculation, taking into account an assumed drop-out rate if applicable. | We appreciate this comment and agree that it is important to clearly state how the sample size of 400 was derived. We have now drafted a new “sample size” section within the methods and outlined how we derived a total study sample size of 400. |
| While the written text appears to be sound, the captions of the figure and table are unclear or incorrectly used, making it difficult to understand the figure and table on its own. I would suggest using a more thorough and complete caption that really describes what the figure or table is about. For instance for the figure I would suggest using something like "Illustration of the study design". For the table, I would suggest using a caption like "Outcomes collected in the study". In addition, although most of the outcomes seem to be included in the table and the table is of added value to the manuscript, the table fails to give an immediately clear overview when certain outcomes are collected. I would advise doing something like this to make it immediately and easily clear to the reader: | We have now implemented a global change of all figure and table labeling. We have also simplified visual for the time periods for study instrument assessment. |
| I believe some collected outcomes are missing in the table (e.g. STI testing). In the text of the manuscript, nothing is being said about the evaluation of psychosocial health outcomes, but you are collecting information about this. The authors should include more detail about why this information is collected. | This has now been added to the revised manuscript. |
| Although the authors clearly describe the inclusion criteria, the exclusion criteria remain unclear and should be incorporated in the manuscript. | Exclusion criteria has now been added to the revised manuscript |
| I am not a statistician, so I can not completely review the statistical analyses you have planned. However, I believe some valuable information is missing about interim or subgroup analysis. Are the authors planning to do so or why not. And if yes, why would the authors do that. | We have updated the data analysis section to be more thorough and specific. |

**Reviewer #5 Comments**

| Comments | Response |
| --- | --- |
| The manuscript includes a strong introduction and literature review. The epidemiological data presented is clear, timely, and nuanced. In particular, the authors note the dearth of current scholarship focused on HCV burden among intersectionally marginalized communities, such as Black and Latino sexual minority men. Overall, both the introduction and literature review provide solid justification for the proposed study protocol and empirical study of  HCV among HIV-negative Black and Latino SMM. | We appreciate this positive appraisal of our work. |
| Study sites and target population. While the methods section, including the subsection 'Study Sites and Target Population,' is well-crafted, in describing the ongoing collaborative partnership between the two key community-based, non-profit organizations, the authors should describe how their partnerships with the community organizations were facilitated. Presently, this is unclear. | We have now provided detailed about how the relationship between Us Helping Us, People into Living Inc (DC) and Abounding Prosperity (Dallas TX) was facilitated |
| Methods: Sample Size. In detailing the proposed protocol study’s research design, the authors should provide an empirical rationale for the proposed study sample size (N = 400). At present, no clear justification is provided. | We appreciate this comment and agree that it is important to clearly state how the sample size of 400 was derived. We have now drafted a new “sample size” section within the methods and outlined how we derived a total study sample size of 400. |
| The reviewer strongly suggests that the authors provide an empirical rationale for the stated inclusion/exclusion criteria related to age (i.e., 18 years and up). Indeed, 18-years and over encompasses a range of developmental stages that may be related to HCV incidence and/or prevalence among the population of interest (BLSMM). As mentioned, please provide an empirical rationale for this methodological relative to other studies that focus on particular age groups, e.g., ages 13 to 24; 18 to 25; 30 and up. Given that the manuscript is a study protocol, it is important for the authors to unpack their methodological decision-making in its entirety. | We have now provided a rationale for the age cutoff utilized in the current study. |
| Notably, the reviewer appreciates the careful attention paid to unpacking this component of the proposed research protocol (i.e., Initial study visit, Integrated STI screening, Study questionnaire, Instruments, Translation of study materials, analysis plan, data management, quality assurance, & control, and statistical analysis). Important given the outcomes and study population | We appreciate this positive appraisal of our work and revisions. |
|  |  |
|  |  |
